# Factorial Analysis of the Cardiometabolic Risk Influence on Redox Status Components in Adult Population

**DOI:** 10.1155/2021/6661940

**Published:** 2021-01-27

**Authors:** Aleksandra Klisic, Nebojsa Kavaric, Sanja Vujcic, Vesna Spasojevic-Kalimanovska, Jelena Kotur-Stevuljevic, Ana Ninic

**Affiliations:** ^1^Primary Health Care Center, University of Montenegro-Faculty of Medicine, Podgorica, Montenegro; ^2^Department for Medical Biochemistry, University of Belgrade-Faculty of Pharmacy, Belgrade, Serbia

## Abstract

Different byproducts of oxidative stress do not always lead to the same conclusion regarding its relationship with cardiometabolic risk, since controversial results are reported so far. The aim of the current study was to examine prooxidant determinant ((prooxidant-antioxidant balance (PAB)) and the marker of antioxidant defence capacity (total sulphydryl groups (tSHG)), as well as their ratio (PAB/tSHG) in relation to different cardiometabolic risk factors in the cohort of adult population. Additionally, we aimed to examine the joint effect of various cardiometabolic parameters on these markers, since to our knowledge, there are no studies that investigated that issue. A total of 292 participants underwent anthropometric measurements and venipuncture procedure for cardiometabolic risk factors assessment. Waist-to-height ratio (WHtR), body mass index, visceral adiposity index (VAI), and lipid accumulation product (LAP) were calculated. Principal component analysis (PCA) grouped various cardiometabolic risk parameters into different factors. This analysis was used in the subsequent binary logistic regression analysis to estimate the predictive potency of the factors towards the highest PAB and tSHG values. Our results show that triglycerides, VAI, and LAP were positively and high density lipoprotein cholesterol (HDL-c) were negatively correlated with tSHG levels and vice versa with PAB/tSHG index, respectively. On the contrary, there were no independent correlations between each cardiometabolic risk factor and PAB. PCA revealed that obesity-renal function-related factor (i.e., higher WHtR, but lower urea and creatinine) predicts both high PAB (OR = 1.617, 95% CI (1.204-2.171), *P* < 0.01) and low tSHG values (OR = 0.443, 95% CI (0.317-0.618), *P* < 0.001), while obesity-dyslipidemia-related factor (i.e., lower HDL-c and higher triglycerides, VAI, and LAP) predicts high tSHG values (OR = 2.433, 95% CI (1.660-3.566), *P* < 0.001). In conclusion, unfavorable cardiometabolic profile was associated with higher tSHG values. Further studies are needed to examine whether increased antioxidative capacity might be regarded as a compensatory mechanism due to free radicals' harmful effects.

## 1. Introduction

A growing body of evidence revealed an enhanced prooxidant environment in a variety of cardiometabolic disorders. At the same time, the antioxidative potential of many enzymatic and nonenzymatic biomolecules is compromised in an attempt to cope with increased free radicals production [1–3].

Free radicals, if not properly and timely scavenged or decomposed by antioxidants, destroy cellular functionality and structures including lipid membranes, proteins, and nucleic acids and even lead to cellular death [1, 2, 4]. Since reactive oxygen/nitrogen species (ROS/RNS) are highly reactive and exhibit a short half-life, their measurement is difficult [4]. Therefore, oxidative stress secondary products are measured for such purposes. However, different byproducts of oxidative stress do not always reveal the same conclusion regarding its relationship with cardiometabolic risk. Hence, there is no universal index by which oxidative stress can be defined [5].

Prooxidant-antioxidant balance (PAB) represents the ratio of prooxidants to antioxidants concomitantly in one assay, which is calibrated on the basis of different ratios of uric acid and hydrogen peroxide [6, 7]. High serum PAB values are assumed to be related to an increased production of ROS/RNS. It is presumed that PAB can be a better determinant of oxidative stress than each prooxidant measured separately [7].

Total protein sulphydryl groups (tSHG), a part of the antioxidant system also called thiols, are derived from amino acids, such as methionine and cysteine and their derivatives (i.e., homocysteine and glutathione), both in extracellular fluids and cells [8, 9]. In the state of increased ROS/RNS production, tSHG could be oxidized, and therefore, the antioxidant defence pool becomes diminished. Hence, the serum levels of tSHG directly represent the whole-body redox status, in terms that its decrease is indicative of increased oxidative stress, and vice versa its higher levels are attributed to cell repairment and detoxification of the harmful effects of ROS/RNS [2]. Namely, in case of increased prooxidant milieu, tSHG becomes oxidized and converted into disulphide bond structures. This process is reversible, and when prooxidant environment resolves, disulphide bonds shift into reduced thiol groups, therefore maintaining a thiol/disulphide and extracellular redox homeostasis [2, 8–10].

Although the majority of studies reported increased prooxidant byproducts and decreased antioxidative molecules and antioxidative enzyme activities [11–17] in many diseases, others reported the opposite, i.e., no changes or even increased antioxidative capacity [18–22].

As far as we know, there are no studies that investigated the joint effect of various cardiometabolic parameters on oxidative stress status. In the light of all these facts, the relationship between the antioxidant defence system and cardiometabolic disturbances still represents an open question. Therefore, in the current study, we aimed to examine PAB as a determinant of an increased prooxidant milieu and a marker of antioxidant defence capacity (tSHG), as well as their ratio (PAB/tSHG index) in relation to different cardiometabolic risk factors in the cohort of adult population. Moreover, to better understand the influence of different cardiometabolic risk factors on high PAB and tSHG, we aimed to group them into several factors in line with their common pathophysiological characteristics and further examine the direction of such potential relationships.

## 2. Patients and Methods

### 2.1. Subjects

A cohort of 292 patients participated in this cross-sectional study, which was approved by the Institutional Ethics Committee of the Primary Health Care Center, Podgorica, Montenegro. The patients were recruited consecutively in the period from May to July 2017. Each participant provided signed informed consent, with an attached filled questionnaire consisting of answers regarding demographic data, lifestyle habits, and acute/chronic diseases.

Blood pressure (systolic (SBP) and diastolic blood pressure (DBP)) and anthropometric indices (body weight (kg), height (cm), and waist circumference (WC)) were provided for each participant, whereas waist-to-height ratio (WHtR) and body mass index (BMI) were calculated. Lipid indices were calculated also [23].

Visceral adiposity index (VAI) was obtained as follows: {[WC/36.58 + (1.89 × BMI)] × (TG/0.81) × (1.52/HDL − c)} for women, and {[WC/39.68 + (1.88 × BMI)] × (TG/1.03) × (1.31/HDL − c)} for men, where WC is expressed in cm, BMI in kg/m^2^, and triglycerides (TG) and high density lipoprotein cholesterol (HDL-c) in mmol/L.

Lipid accumulation product (LAP) was provided as follows: [(WC − 58) x TG] for women and [(WC − 65) x TG] for men, where WC is expressed in cm and TG in mmol/L.

In addition to lipid indices, we have also calculated siMS score, as a novel comprehensive approach for metabolic status quantification [24]. It was calculated as follows: siMS score = 2 x WC/height + glucose/5.6 + TG/1.70 + SBP/130–HDL − c/1.02 or 1.28 (for males and females, respectively), where WC and height are expressed in cm, glucose, TG, and HDL-c in mmol/L and SBP in mmHg [24].

The inclusion criteria for the current study were participants older than 18 years and who were willing to enter the study. On the other hand, subjects that reported acute infection, endocrine disorders other than type 2 diabetes, malignant disease, severe anaemia, acute myocardial infarction or stroke in the previous 6 months, ethanol consumption >20 g/day, use of antibiotics, glucocorticoids and nonsteroidal anti-inflammatory medications, and pregnancy were excluded from the study. Additionally, patients with an estimated glomerular filtration rate (eGFR_MDRD_) < 30 mL/min/1.73 m^2^ and with high-sensitivity C-reactive protein (hsCRP) (>10 mg/L) were also excluded.

### 2.2. Methods

Two blood samples were obtained from each individual. The venipuncture was performed in the morning after a fast of at least 8 hours. The preanalytical processes of blood sampling and analyses have been described previously [12]. In brief, one blood sample was provided in the tube with serum separator and clot activator for determination of cardiometabolic and oxidative stress parameters, whereas the other one was collected in the tube with K_2_EDTA for measurement of glycated haemoglobin (HbA1c) levels.

All biochemical parameters (i.e., fasting glucose, HbA1c, hsCRP, creatinine, urea, uric acid, gamma glutamyl transferase (GGT), total cholesterol, TG, HDL-c, and low-density lipoprotein cholesterol (LDL-c)) were analyzed using Roche Cobas c501 chemistry analyzer (Roche Diagnostics GmbH, Mannheim, Germany), using standard procedures.

Furthermore, tSHG groups were determined spectrophotometrically using 5,5'-dithiobis (2- nitro benzoic acid) [25], whereas PAB measurement was done spectrophotometrically using 3,3', 5,5'-tetramethylbenzidine as a chromo gen [6].

## 3. Statistical Analysis

Data distributions were tested by the Kolmogorov-Smirnov test. Normally distributed data were compared by Student's *t*-test and presented as arithmetic mean ± standard deviation. Skewed data were compared by the Mann–Whitney test and presented as median (interquartile range). Categorical variables were compared by Chi-square test for contingency tables and given as absolute and relative frequencies. Correlation coefficient (*ρ*) determination was performed by nonparametric bivariate Spearman's correlation analysis. To assess in-depth associations of clinical markers with tSHG, PAB levels and PAB/tSHG index (ordinal dependent variables given as tertiles) in univariate and multivariate ordinal regression analysis were applied. In univariate analysis, the independent variables were the indices VAI and LAP, and anthropometric (BMI and WC) or lipid markers (HDL-c and TG) were used for their calculations. They had the identical effect at each cumulative split of each ordinal dependent variable. In multivariate analysis, covariates were selected based on these criteria: firstly, continuous variables were in significant bivariate correlation with the dependent variable, and secondly, categorical variables had an unequal significant distribution between ordinal dependent variable tertiles. In multivariate ordinal regression analysis, none of the covariates showed multicollinearity. Data from ordinal regression analysis were presented as odds ratios (ORs) and 95% confidence intervals (CIs). The explained variations in tSHG, PAB levels, and PAB/tSHG index in the observed population were given by the Nagelkerke *R*^2^ value. Principal component analysis (PCA) [26] with varimax–normalized rotation was used to reduce the number of variables (which were in significant correlation with tSHG, PAB, and PAB/tSHG index) in several significant factors. Criterion for factor extraction was its >1, and factor loadings >0.5, and both were used for variable inclusion; the number of factors was fixed at 3. PCA produced scores for significant factors, which were used in the subsequent binary logistic regression analysis to estimate the predictive potency of the factors towards the highest PAB and tSHG values. The IBM® SPSS® Statistics version 22 software (USA) was used for statistical calculations. Significance level set at *P* value less than 0.05 was considered statistically significant.

## 4. Results

Demographic and clinical characteristics of the examined population were compared between men and women and were listed in Table 1. All participants were of similar ages. Although males and females did not differ in BMI and WHtR, females had lower WC than males. More patients with type 2 diabetes were among males than females, as well as more antihyperglycemic, insulin, and antihypertensive users were among males.

Males had higher glucose, HbA1c, GGT, uric acid levels, and tSHG levels than females (Table 2). However, females had higher lipid status markers (TC, HDL-c, and LDL-c), PAB levels, and PAB/tSHG index than males (Table 2). TG and hsCRP levels, VAI and LAP indices, and siMS score did not show significant differences between genders.

Since there were significant differences between oxidative stress markers (tSHG, PAB, and PAB/tSHG index) between males and females, we further wanted to examine whether or not anthropometric and/or lipid markers could serve as their potential determinants. Firstly, we performed Spearman's correlation analysis (Table 3). tSHG positively correlated with WC, WHtR, glucose, HbA1c, TG, GGT, uric acid, VAI, LAP, and siMS score. Significant negative correlation was only evident between tSHG and HDL-c. PAB levels correlated negatively with age, glucose, HbA1c, GGT, and uric acid and positively with SBP, HDL-c, LDL-c, and hsCRP. PAB/tSHG index demonstrated mostly negative correlations with age, WC, glucose, HbA1c, TG, GGT, uric acid, VAI, LAP, and siMS score. Positive correlation was observed between PAB/tSHG index and HDL-c.

Further investigations of the associations between tSHG, PAB levels, and PAB/tSHG index and anthropometric (BMI and WC), lipid (HDL-c and TG) markers and their indices (VAI and LAP), and siMS score were assessed by ordinal regression analysis (Table 4).

In univariate ordinal regression analysis, WC, TG, VAI, LAP, and siMS score positively and HDL-c negatively correlated with tSHG (Table 4). Examinees with higher WC, TG, VAI, LAP, and siMS score were 1.029, 2.337, 1.560, 1.015, and 2.020 times, respectively, more likely to exhibit higher tSHG levels. However, the odds of having higher tSHG was 72% greater in participants with lower HDL-c. Nagelkerke *R*^2^ for WC, HDL-c, TG, VAI, LAP, and siMS score were 0.038, 0.086, 0.170, 0.158, 0.144, and 0.148, respectively, which means that 3.8%, 8.6%, 17%, 15.8%, 14.4%, 14.8%, of variation in tSHG could be explained by each marker. Before performing multivariate ordinal regression analysis, we tested categorical data distribution among tSHG tertiles to determine potential covariates. Unequal distributions were evident for gender (*P* = 0.003), diabetes presence (*P* = 0.036), antihyperglycemic (*P* = 0.003), insulin (*P* = 0.003), and antilipemic (*P* = 0.035) users between tSHG tertiles.

Independent continuous variables which correlated significantly with tSHG (Table 3) (HbA1c, GGT, and uric acid) and categorical variables (diabetes presence, antilipemic therapy, and gender) obtained by Chi-square analysis were included in the multivariate ordinal regression analysis as covariates (Table 4). TG, VAI, and LAP kept their positive and HDL-c negative independent associations and predictions of tSHG levels.

In univariate ordinal regression analysis, only HDL-c was positively associated with PAB levels (Table 4). Odds of having higher PAB levels were 1.976 times greater in participants with higher HDL-c concentration. Nagelkerke *R*^2^ was 0.027, indicating that 2.7% variation in PAB levels could be explained by HDL-c (Table 4). When tested categorical data distribution among PAB levels tertiles, we determined unequal distributions for gender (*P* < 0.001), diabetes presence (*P* = 0.001), antihyperglycemic (*P* = 0.004), antihypertensive (*P* = 0.006) users between tertiles. Independent continuous variables which correlated significantly with PAB levels (Table 3) (age, HbA1c, hsCRP, GGT, and uric acid) and categorical variables (diabetes presence, antihypertensive therapy, and gender) obtained by Chi-square analysis were included in the multivariate ordinal regression analysis as covariates (Table 4). HDL-c lost significant independent association and prediction of PAB levels.

In univariate ordinal regression analysis, WC, TG, VAI, LAP, and siMS score were negatively and HDL-c positively associated with PAB/tSHG index (Table 4). Examinees with lower WC, TG, VAI, LAP, and siMS score were 2.2%, 25.8%, 21.6%, 0.8%, and 39.7%, respectively, more likely to exhibit higher PAB/tSHG index. However, the odds of having the higher PAB/tSHG index were 3.699 times greater in participants with higher HDL-c. Nagelkerke *R*^2^ for WC, HDL-c, TG, VAI, LAP, and siMS score were 0.024, 0.088, 0.088, 0.075, 0.062, and 0.090, respectively, which means that 2.4%, 8.8%, 8.8%, 7.5%, 6.2%, and 9.0% of variation in PAB/tSHG index could be explained by each marker. When tested categorical data distribution among PAB/tSHG tertiles, we determined unequal distributions for gender (*P* < 0.001), diabetes presence (*P* = 0.002), antihyperglycemic (*P* < 0.001), antihypertensive (*P* = 0.032), and antilipemic (*P* = 0.033) users between tertiles.

Independent continuous variables which correlated significantly with PAB/tSHG index (Table 3) (age, HbA1c, GGT, and uric acid) and categorical variables (diabetes presence, antihypertensive and antilipemic therapy and gender) obtained by Chi-square analysis were included in the multivariate ordinal regression analysis as covariates (Table 4). TG, VAI, and LAP kept their negative and HDL-c positive independent associations and predictions of PAB/tSHG index.

PCA was implemented to get a smaller number of factors grouped according to the same level of variability, from the large number of parameters which significantly correlated with tSHG and PAB. This analysis emphasized 3 different factors explaining 72% of variance of the tested parameters (Table 5). The largest percent of variance (43%) showed the first, obesity-dyslipidemia-related factor with positive loadings of TG and lipid indices (VAI and LAP) and with negative loading of HDL-c. The second factor explained 16% of the variation and consisted of obesity-renal function parameters (obesity with positive and renal factors with negative loadings), and the third, blood pressure-related factor, explained 13% of the variation (both parameters with positive loadings).

Binary logistic regression analysis enabled us to estimate which factors, expressed as scores and given by PCA, could predict high PAB and tSHG values (Table 6). Our analysis showed that obesity-renal function-related factor predicts both high PAB and low tSHG, while obesity-dyslipidemia-related factor predicted significantly only high tSHG values. The third factor (i.e., blood pressure-related factor) did not predict either PAB or tSHG values.

## 5. Discussion

This study has shown several findings that merit to be emphasized. Firstly, tSHG and PAB/tSHG indices were independently correlated with lipid parameters (HDL-c and TG) and lipid indices (VAI and LAP), whereas no independent correlation between PAB and each examined parameter alone was found. Namely, multivariate ordinal regression analysis revealed that HDL-c showed negative and TG, VAI, and LAP positive independent associations and predictions of tSHG and vice versa of PAB/tSHG levels. Secondly, PCA analysis after grouping the variables into three factors (i.e., obesity-dyslipidemia related factor, obesity-renal function-related factor, and blood pressure-related factor) produced PCA scores for the mentioned factors. These scores were used in binary logistic regression analysis to estimate its predictive ability towards high PAB and tSHG values. Obesity-renal function-related factor (which included higher WHtR, but lower urea and creatinine) predicted high PAB values, but at the same time low tSHG values. The direction of this influence was opposite, i.e., higher values of this factor increase PAB, but a lower summary value of the same factor predicted higher tSHG. Obesity-dyslipidemia-related factor (which included lower HDL-c and higher TG, VAI, and LAP) predicted higher tSHG values, and this could be explained as a compensatory induction of available antioxidative potential. On the contrary, the third factor connected with blood pressure homeostasis did not predict any of these two redox status-related parameters. Such findings represent the novelty, since to the best of our knowledge, there are no studies that examined the joint effect of various cardiometabolic parameters on the circulating levels of prooxidants and antioxidants. Moreover, no previous studies examined lipid indices (VAI and LAP) in relation to oxidative stress indicators in the adult population. We have also shown for the first time positive association between siMS score and tSHG, and an inverse association between siMS and PAB/tSHG index, respectively. The siMS score represents a novel comprehensive score for quantification of metabolic status that included beside body height, several metabolic syndrome related parameters, i.e., WC, glucose, TG, and HDL-c, as well as SBP in its calculation) [24]. Furthermore, the relatively large sample size of our study (i.e., nearly 300 participants) is another strength of our research.

Previous reports suggested VAI and LAP as better determinants of cardiometabolic risk than anthropometric measures (i.e., BMI and WC) [27]. Indeed, although anthropometric indices correlated with oxidative stress determinants, these correlations lost their independence after further analysis in our study. Since both of these lipid indices are gender-specific and include a simple measure of central obesity as a principal indicator of increased cardiometabolic risk, it would be expected that with a greater extent of central obesity, prooxidants would be increased, whereas antioxidants would be lowered, as previous studies reported [3, 11]. However, results in the current study have shown the opposite, such as positive association between lipid indices and tSHG. Enhancement of antioxidant protection (in terms of higher tSHG) may be attributed to its compensation due to increased free radicals generation, since in the obese state prooxidant-antioxidant homeostasis is disturbed [3].

In such circumstances, lipids become very susceptible to oxidation. Enlarged visceral adipose tissue contributes to lipid accumulation and lipid peroxidation. The increased production of highly reactive free radicals (i.e., superoxide anions) through the mitochondrial electron transport chain can cause cellular damage in almost all organs if the antioxidant defence system fails to cope with them, as previously stated [1, 4, 28].

In a nutshell, proinflammatory adipokines (i.e., leptin, resistin, visfatin, apelin, etc.) and cytokines (interleukin-1, interleukin-6, tumor necrosis factor alpha, etc.), which are secreted by the enlarged visceral adipose tissue, affect insulin signalling pathways favouring the insulin resistant state. Therefore, antilipolytic effects of insulin and phosphatidylinositol 3-kinase (PI3K) pathways are compromised in favour of increased lipolysis of TG and increased release of free fatty acids (FFA) from adipose tissue. The latter reach the liver, enhancing oxidative phosphorylation, ROS production, and liver fat peroxidation [3], leading to increased synthesis of NADH/NAD+ ratio in mitochondria. All these processes favour an enhanced activation of protein kinase C (PKC), increased activity of nicotinamide adenine dinucleotide phosphate (NADPH) oxidase, and increased free radicals production. Consequently, inhibition of endothelial nitric oxide synthase (eNOS) and decreased nitric oxide (NO) synthesis, a potent vasodilatator, in vascular smooth muscle cells occur and precede vasoconstriction and endothelial dysfunction [3, 4].

The increased hepatic synthesis of lipotoxic TG-rich very-low-density lipoproteins (VLDL), small dense low-density lipoproteins (sdLDL), and shifted distribution of HDL particles to smaller proatherogenic HDL3 ones is another consequence of insulin resistant state [29]. The sdLDL and smaller HDL3 particles are susceptible to oxidative modifications and enable the onset and progression of atherosclerosis [30]. Moreover, cytotoxic effects of FFA and accumulated lipids lead to other organ impairments, such as tubulointerstitial and glomerular cells injury, thus promoting renal disease [31]. In line with this, the independent association between renal function markers and lipid parameters was reported earlier [32, 33].

Additionally, it was reported that sterol regulatory element-binding protein-1c (SREBP1-c), a sort of transcription factor, is involved in the regulation of gene expression responsible for the differentiation of adipocytes, lipogenesis, and FFA oxidation [3].

Since ROS/RNS are generated through various pathways, each prooxidant and antioxidant represent a different measure of oxidative stress [5]. This could explain in part the discrepancies in the results concerning antioxidative defence capacity in various cardiometabolic disorders. A study by Pande et al. [34] has shown higher levels of total thiol (i.e., tSHG) in both patients with prediabetes and diabetes as compared to control group. On the contrary, no difference in serum tSHG levels was reported between women with IR and noninsulin-resistant counterparts [18], as well as in patients with diabetes and prediabetes vs. control group [16]. Additionally, differences in ethnic background, sample sizes, and gender distribution in the examined cohorts and even different methods for measurement of prooxidant and antioxidant markers may also contribute to the inconclusive results. Moreover, the degree and duration of obesity and other comorbidities might be an important bias factor since antioxidative enzymes might be enhanced at the beginning of the process, but later depleted when the antioxidative pool becomes exhausted [3, 20]. Our unexpected results might also be explained by other environmental factors such as regular physical activity and nutritional habits (dietary factors) which may increase synthesis of antioxidant molecules and induce antioxidative enzymes activity [4, 35]. Namely, adequate intake of proteins and supplement N-acetylcysteine (which contains thiol compounds) may lead to the increase in tSHG [36]. Also, daily intake of antioxidant vitamins [4] may significantly contribute to cellular redox homeostasis. Unfortunately, this study is limited for such information. The other limitation of our study is its cross-sectional nature, and thus, the causality between the unfavorable cardiometabolic profile and higher tSHG and PAB could not be confirmed. At last, we were limited to examine thiol-disulphide homeostasis, which could provide better insight into the whole-body redox status [8].

## 6. Conclusion

Unfavorable cardiometabolic profile was associated with higher tSHG values. Longitudinal design of other studies is needed to further explore the underlying mechanisms of the relationship between prooxidants, antioxidants, and cardiometabolic disturbances and to enlighten the role of antioxidants in the fight against free radicals production and its negative side effects. These findings would enable us to find the most appropriate pharmaceutical target for treatment of population with increased cardiometabolic risk.

## Figures and Tables

**Table 1:** General data of participants according to gender.

	Men	Women	*P*
*N*	110	182	
Age, years	63 (56-68)	61 (56-69)	0.878
BMI, kg/m^2^	29.2 (26.9-32.0)	28.2 (25.1-31.6)	0.082
WC, cm^#^	104.5 ± 9.8	95.4 ± 11.1	<0.001
WHtR	0.587 ± 0.054	0.582 ± 0.071	0.528
SBP, mmHg	134 (126-144)	133 (124-148)	0.642
DBP, mmHg	85 (78-90)	84 (77-94)	0.923
Diabetes, %	49.1	26.9	<0.001
Diabetes duration, years	6 (2-10)	4 (1-10)	0.251
Smokers, %	21.8	18.9	0.515
Antihyperglycemics, %	42.7	23.1	<0.001
Insulin therapy, %	16.4	7.7	0.022
Antihypertensives, %	71.8	60.4	0.049
Hypolipidemics, %	38.2	31.9	0.271

Data are presented as median (interquartile range) and compared with the Mann–Whitney *U*-test. ^#^Normally distributed data are presented as arithmetic mean ± standard deviation and compared with Student's *t*-test. Categorical variables are presented as relative frequencies and compared by Chi-square test for contingency tables.

**Table 2:** Laboratory markers of the examined population according to gender.

	Men	Women	*P*
Glucose, mmol/L	6.1 (5.5-8.2)	5.7 (5.3-6.7)	0.002
HbA1c, %	5.9 (5.5-7.2)	5.6 (5.3-6.2)	<0.001
Total cholesterol, mmol/L	5.09 (4.57-6.03)	6.06 (5.13-6.88)	<0.001
HDL-c, mmol/L	1.20 (0.98-1.43)	1.44 (1.19-1.76)	<0.001
LDL-c, mmol/L	3.02 (2.36-3.84)	3.68 (2.78-4.40)	<0.001
TG, mmol/L	1.81 (1.36-2.44)	1.85 (1.20-2.54)	0.714
HsCRP, mg/L	1.05 (0.53-2.38)	1.18 (0.59-2.29)	0.804
GGT, U/L	22 (16-33)	15 (11-21)	<0.001
Uric acid, *μ*mol/L	330 (277-383)	265 (215-318)	<0.001
VAI	2.20 (1.41-3.29)	1.77 (1.08-3.21)	0.137
LAP	73.40 (46.50-98.90)	65.22 (36.40-99.45)	0.254
tSHG, *μ*mol/L	0.29 (0.24-0.35)	0.24 (0.19-0.31)	<0.001
PAB, HKU	95.85 (61.30-115.10)	116.05 (74.10-136.00)	<0.001
PAB/tSHG index	314 (213-439)	464 (304-644)	<0.001
siMS score	3.46 (2.78-3.99)	3.22 (2.49-3.86)	0.243

Data are presented as median (interquartile range) and compared with the Mann–Whitney *U*-test.

**Table 3:** The bivariate Spearman's correlation analysis between tSHG, PAB, and PAB/tSHG index and clinical markers.

	tSHG, *μ*mol/L	PAB, HKU	PAB/tSHG index
Age, years	-0.037	-0.215∗∗∗	-0.121∗
BMI, kg/m^2^	0.092	-0.059	-0.093
WC, cm	0.183∗∗	-0.084	-0.164∗∗
WHtR	0.158∗∗	0.036	0.017
SBP, mmHg	-0.005	0.116∗	0.072
DBP, mmHg	0.004	0.035	0.016
Glucose, mmol/L	0.243∗∗∗	-0.192∗∗	-0.294∗∗∗
HbA1c, %	0.271∗∗∗	-0.138∗	-0.237∗∗∗
Total cholesterol, mmol/L	-0.029	0.160∗∗	0.114∗
HDL-c, mmol/L	-0.355∗∗∗	0.116∗	0.308∗∗∗
LDL-c, mmol/L	-0.061	0.178∗∗	0.146∗
TG, mmol/L	0.397∗∗∗	-0.096	-0.318∗∗∗
HsCRP, mg/L	0.033	0.154∗∗	0.067
GGT, U/L	0.258∗∗∗	-0.150∗	-0.265∗∗∗
Uric acid, *μ*mol/L	0.171∗∗	-0.206∗∗∗	-0.258∗∗∗
VAI	0.391∗∗∗	-0.104	-0.334∗∗∗
LAP	0.347∗∗∗	-0.094	-0.320∗∗∗
tSHG, *μ*mol/L	—	-0.114	-0.742∗∗∗
PAB, HKU	-0.114	—	0.701∗∗∗
siMS score	0.377∗∗	-0.111	-0.321∗∗

Data are presented as the correlation coefficient Rho (*ρ*). ∗*P* < 0.05, ∗∗*P* < 0.01, ∗∗∗*P* < 0.001.

**Table 4:** Estimated odds ratios after ordinal regression analysis for tSHG, PAB, and PAB/tSHG tertiles, respectively, as dependent variable.

Unadjusted			
*tSHG tertiles as dependent variable*	*OR (95% CI)*	*P*	*NagelkerkeR* ^2^
BMI	1.046 (0.957-1.097)	0.072	0.013
WC	1.029 (1.011-1.049)	0.002	0.038
WHtR	2.27 (2.19-2.37)	0.022	0.037
HDL-c	0.281 (0.164-0.481)	<0.001	0.086
TG	2.337 (1.784-3.062)	<0.001	0.170
VAI	1.560 (1.339-1.820)	<0.001	0.158
LAP	1.015 (1.010-1.020)	<0.001	0.144
siMS score	2.020 (1.600-2.550)	<0.001	0.148
Adjusted			
*Model*	*OR (95% CI)*	*P*	*NagelkerkeR* ^2^
WC	1.011 (0.989-1.033)	0.319	0.088
WHtR	0.128 (0.065-0.325)	0.069	0.121
HDL-c	0.359 (0.199-0.646)	0.001	0.126
TG	2.333 (1.754-3.102)	<0.001	0.220
VAI	1.540 (1.310-1.811)	<0.001	0.204
LAP	1.015 (1.009-1.021)	<0.001	0.192
Unadjusted			
*PAB tertiles as dependent variable*	*OR (95% CI)*	*P*	*NagelkerkeR* ^2^
BMI	0.978 (0.932-1.026)	0.371	0.013
WC	0.985 (0.967-1.003)	0.104	0.010
HDL-c	1.976 (1.185-3.294)	0.009	0.027
TG	0.924 (0.768-1.112)	0.401	0.002
VAI	1.001 (0.924-1.083)	0.988	0
LAP	0.999 (0.996-1.003)	0.677	0.001
siMS score	0.840 (0.694-1.017)	0.075	0.012
Adjusted			
*Model*	*OR (95% CI)*	*P*	*NagelkerkeR* ^2^
HDL-c	1.142 (0.640-2.056)	0.653	0.136
Unadjusted			
*PAB/tSHG tertiles as dependent variable*	*OR (95% CI)*	*P*	*NagelkerkeR* ^2^
BMI	0.970 (0.924-1.019)	0.217	0.006
WC	0.978 (0.959-0.995)	0.014	0.024
HDL-c	3.699 (2.145-6.360)	<0.001	0.088
TG	0.742 (0.467-0.749)	<0.001	0.088
VAI	0.784 (0.691-1.124)	<0.001	0.075
LAP	0.992 (0.987-0.996)	<0.001	0.062
siMS score	0.603 (0.486-0.748)	<0.001	0.090
Adjusted			
*Model*	*OR (95% CI)*	*P*	*Nagelkerke*R^2^
WC	1.014 (0.991-1.039)	0.231	0.164
HDL-c	2.206 (1.214-4.011)	0.009	0.181
TG	0.602 (0.466-0.788)	<0.001	0.216
VAI	0.810 (0.709-0.925)	0.002	0.200
LAP	0.993 (0.988-0.998)	0.010	0.185

Data are given as OR (95% CI). Adjusted model for tSHG: Model included each marker and continuous (HbA1c, GGT, and uric acid) and categorical variables (diabetes presence, antilipemic therapy, and gender). Adjusted model for PAB: Model included continuous variables: age, HbA1c, hsCRP, GGT, uric acid, and categorical variables: diabetes presence, antihypertensive therapy, and gender. Adjusted model for PAB/tSHG: Model included continuous variables: age, HbA1c, GGT, uric acid, and categorical variables: diabetes presence, antihypertensive and antilipemic therapy, and gender.

**Table 5:** Principal component analysis extracted factors connected with tSHG and PAB values.

Factors	Included variables with loadings	Factor variability
Obesity-dyslipidemia related factor	HDL-c (-0.628)	43%
	TG (0.956)	
	VAI (0.964)	
	LAP (0.959)	
Obesity-renal function-related factor	WHtR (0.581)	16%
	Urea (-0.652)	
	Creatinine (-0.666)	
Blood pressure-related factor	SBP (0.884)	13%
	DBP (0.903)	

**Table 6:** Binary logistic regression analysis of the highest PAB and the highest tSHG tertile values.

Predictors	PAB values (3^rd^ tertile)	tSHG values (3^rd^ tertile)
Obesity-dyslipidemia-related factor	1.069 (0.821-1.391)	2.433 (1.660-3.566)∗∗∗
Obesity-renal function-related factor	1.617 (1.204-2.171)∗∗	0.443 (0.317-0.618)∗∗∗
Blood pressure-related factor	1.184 (0.889-1.576)	1.013 (0.763-1.345)

Data are presented as OR (95% CI). Abbreviations: OR, odds ratio; CI, confidence interval. ∗∗*P* < 0.01, ∗∗∗*P* < 0.001.

## Data Availability

The data will be available upon reasonable request (contact person: aleksandranklisic@gmail.com).
